# Population pharmacokinetics of levodopa/carbidopa microtablets in healthy subjects and Parkinson’s disease patients

**DOI:** 10.1007/s00228-018-2497-2

**Published:** 2018-06-07

**Authors:** Marina Senek, Dag Nyholm, Elisabet I. Nielsen

**Affiliations:** 1 Department of Neuroscience, Neurology, Uppsala University, Akademiska Sjukhuset/Uppsala University Hospital, 751 85 Uppsala, Sweden; 2 Department of Pharmaceutical Biosciences, Uppsala University, Husarg. 3, Box 591, 751 24 Uppsala, Sweden

**Keywords:** Parkinson’s disease, Population modeling, Levodopa, Microtablets, Pharmacokinetics

## Abstract

**Objectives:**

Low dose, dispersible, levodopa/carbidopa microtablets with an automatic dose dispenser have been developed to facilitate individualized levodopa treatment. The aim of this study was to characterize the pharmacokinetics (PK) of levodopa and carbidopa after microtablet administration, and evaluate the impact of potential covariates.

**Methods:**

The population PK analysis involved data from 18 healthy subjects and 18 Parkinson’s disease patients included in two single-dose, open-label levodopa/carbidopa microtablet studies. The analysis was carried out using non-linear mixed effects modeling. Bodyweight was included on all disposition parameters according to allometric scaling. Potential influence of additional covariates was investigated using graphical evaluation and adjusted adaptive least absolute shrinkage and selection operator.

**Results:**

Dispositions of levodopa and carbidopa were best described by a two- and one-compartment model respectively. Double-peak profiles were described using two parallel absorption compartments. Levodopa apparent clearance was found to decrease with increasing carbidopa dose (15% lower with 75 compared to 50 mg of carbidopa) and disease stage (by 18% for Hoehn and Yahr 1 to 4). Carbidopa apparent clearance was found to decrease with age (28% between the age of 60 and 80 years). An external evaluation showed the model to be able to reasonably well predict levodopa concentrations following multiple-dose microtablet administration in healthy subjects.

**Conclusions:**

The presented models adequately described the PK of levodopa and carbidopa, following microtablet administration. The developed model may in the future be combined with a pharmacokinetic-pharmacodynamic target and used for individualized dose selection, utilizing the flexibility offered by the microtablets.

**Electronic supplementary material:**

The online version of this article (10.1007/s00228-018-2497-2) contains supplementary material, which is available to authorized users.

## Introduction

In the initial stage of Parkinson’s disease (PD), the effect from levodopa (LD) and carbidopa (CD) treatment is often satisfactory, with a stable motor function that is near normal throughout the day. As the disease progresses, the treatment effect duration shortens, and as the effect wears off, disease-specific symptoms reappear. With further disease progression, patients may develop involuntary movements (i.e., dyskinesia), typically occurring as a LD peak concentration phenomenon. In this stage, there is a narrow therapeutic window, with a lower threshold for symptom relief, and an upper threshold where dyskinesia appears [1]. The thresholds are individual, and a reason to why tailored doses and dosing intervals are necessary for optimal treatment outcome. For patients with Parkinson’s disease, the pharmacokinetics of LD thus become increasingly important with disease progression.

The LD/CD, low dose, microtablets, 5/1.25 mg [2, 3] (Flexilev®, Sensidose AB, Sollentuna, Sweden), dispensed with an automatic dose dispenser [4] MyFID™ have been developed with the incentive to facilitate individual, frequent dosing of LD/CD treatment. The dispenser records the dosing history (amount and timing) and indicates if a planned dose is missed. It also has a diary function which allows for self-reporting of symptoms that can be viewed by both the patient and the treating physician. This enables easier follow-up of treatment and evaluation of the therapy. The patients can become more involved in their treatment, and for the physicians, it can serve as an aid in therapeutic decision-making. This treatment alternative allows the dosages to be highly individualized, but initial investigations have shown that it can be challenging to find the optimal dosing regimen [5]. A population pharmacokinetic (PK) model for the LD/CD microtablets incorporating influential covariates provides a better understanding of LD’s pharmacokinetics. The model, in combination with a pharmacodynamic model, can be used to visualize the individuals expected time profile of the concentration and effect (size and duration) based on current dosing, and be used to facilitate the tailoring of treatment to the need of individual patients.

This analysis aimed to characterize the pharmacokinetics of LD and CD following LD/CD microtablet administration in patients and healthy subjects, and to explore potential differences between patients with advanced PD and healthy subjects using a population model-based approach.

## Methods

### Study data

Data from two previously published single-center, open-label, single-dose studies were combined and used for the PK model development (Study 1–2) [2, 6]. Additionally, data from a previously published single-center, open-label, multiple-dose study was used for external model evaluation (Study 3) [3]. The bioanalytical methods are described in the supplementary material (Online Resource 1).

#### Study 1

Study 1 included 18 healthy subjects (Table 1) who received 100/25 mg of LD/CD microtablets (i.e., 20 tablets dissolved in a glass of 100 mL water) [2]. Blood samples were obtained before dosing (within 1 h before administration of study drug) every 10 min during the first hour; every 20 min between hour 1 and 2; half-hourly between 2 and 3 h; hourly between 3 and 6 h; and at 8, 10, 12, and 24 h after intake.

**Table 1:** Healthy volunteer and patient demographics, mean ± standard deviation [range]

Subjects	Sex^‡^ (M/F)	Age^‡^ (years)	Body weight	Years since diagnosis	HY^‡^	Years on LD treatment	LD doses (mg)	CD doses (mg)	Last blood sample (min)
Healthy (Study 1)	9/9	26.0 ± 6.2 [19–46]	71.7 ± 11.3 [59–95]	NA	NA	NA	100 ± 0	25 ± 0	1440 ± 0
Patients (Study 2)	13/5	71.4 ± 6.3 [61–82]	75.4 ± 11.0 [55–96]	9.6 ± 6.7 [2–33]	3.2 ± 0.9 [2–5]	9.5 ± 6.7 [2–33]	275 ± 86.3 [110–410]	68.75 ± 21.6 (27.5–102.5)	285.8 ± 86.3 [180–360]
Healthy (Study 3)	4/6	24.7 ± 4.3 [20–32]	71.3 ± 13.7 [52–100]	NA	NA	NA	300 ± 0^†^	75 ± 0	810 ± 0

^†^Total drug dose administered, dosing interval 2.4 h. ^‡^Mean value for adjusted adaptive least absolute shrinkage and selection operator (AALASSO) covariate selection process; sex, 0.389; age, 48.7 years; HY, 1.53. *M*, male; *F*, female; *LD*, levodopa; *CD*, carbidopa; *HY*, Hoehn and Yahr stage; *NA*, not applicable

#### Study 2

Study 2 included 19 patients with Parkinson’s disease (Table 1) experiencing motor fluctuations who received 150% of their individual LD equivalent morning dose of LD/CD microtablets (range 110/27.5–410/102.5 mg, dissolved in a glass of 100 mL water) after an overnight washout period [6]. Blood samples were obtained prior to dosing (within 1 h before administration of study drug), in conjunction with dosing at time 0, and thereafter at 15, 30, 45, 60, 80, 100, 120, 150, 180, 210, 240, 300, and 360 min after dose administration. The patients were allowed to discontinue the study at any time, e.g., if they could no longer remain without additional medication. One patient’s plasma concentration displayed an extreme triple-peak pharmacokinetic profile; therefore, the subject was excluded.

#### Study 3

Study 3 included 10 healthy subjects who received six repeated doses of LD/CD microtablets: 75/18.75 mg as a morning dose and then 45/11.25 mg every 2.4 h after the morning dose (Table 1) [3]. Blood samples were collected 5 min prior to each dose administration and then at 20, 40, 60, and 90 min after intake.

### Model development

#### Base model

The LD and CD models were developed separately and then combined for the covariate analysis. Study 2 included patients with advanced Parkinson’s disease, and despite instructions of an overnight washout, all patients had detectible LD plasma concentrations (average 0.59 ± 0.93, range 0.01–3.41 μg/mL), and eight patients had detectible CD plasma concentrations (average 0.03 ± 0.02, range 0.002–0.066 μg/mL) prior to dose administration. The pre-dose LD plasma concentration was thus higher than the low baseline endogenous LD concentrations reported in literature (0.0076 ± 6.4 μg/mL) [7, 8]. These residual LD and CD concentrations were handled by estimating a typical pre-dose concentration with an associated inter-individual variability and an elimination rate according to the estimated individual terminal slopes (λ_2_) for LD and CD, respectively [9].

Double-peak concentration-time profiles were observed in both patients and healthy, both for LD and CD. In order to describe these profiles, several different absorption models were investigated. Parallel absorption compartments, assuming the total amount to be fractionated into two separate dosing compartments [10], were investigated with separately estimated lag times or transit absorption compartments [11], with and without including a mixture model to account for individual differences in the occurrence of double peaks. Additionally, one empirical model and one semi-mechanistic model describing the double-peak profiles for LD have been previously published, and both approaches were also investigated [12]. The empirical model includes two gastric emptying parameter rates, while the semi-mechanistic model uses a feedback mechanism acting via an effect compartment to link the plasma concentration of LD to the rate of gastric emptying.

One and two compartment disposition models were evaluated, parameterized with apparent volume of central (V_C_/F) and peripheral (V_P_/F) compartment, clearance (CL/F) and inter-compartmental clearance (Q/F). Inter-individual variability was included assuming a log-normal or normal (absorption-related parameters) distribution of structural model parameters.

The fraction administered (fa_1_) to the first dosing compartment was estimated on the logit transformed scale to constrain the parameter between 0 and 1, assuming a normal distribution of the structural parameter. The fraction administered to the second dosing compartment was given by 1 − *fa*_1_.

The residual error model was parameterized as proportional, additive, or a combined proportional and additive error model. Since LD and CD were measured in the same sample, their residual error might be correlated. This was handled by estimating a part of the residual error as being shared between LD and CD [13].

#### Covariate model

Bodyweight was included as a primary covariate on all disposition parameters according to the allometric power model,

$$ P{\theta}_i= TVP{\theta}_1\times {\left(\frac{WT}{70}\right)}^{{P\theta}_2} $$

where *Pθ*_*i*_ is the individual parameter value (CL/F, Q/F, V_C_/F, or V_P_/F), *TVPθ*_1_ is the typical parameter value for an adult of 70 kg, WT is bodyweight, and *Pθ*_2_ is an allometric bodyweight exponent fixed to either 0.75 for CL/F and Q/F or 1 for V_C_/F and V_P_/F [14].

Additional covariates included age, sex, study association, Hoehn and Yahr score (HY, disease stage, set to 0 for healthy subjects), CD dose, 3-O-methyldopa area under the curve (including all measurements, calculated with the trapezoid method), time since symptom onset, time since diagnosis, and years with LD treatment (Table 1).

As an initial step, all covariates were graphically analyzed by plotting covariates versus the empirical Bayes estimates for relevant LD and CD PK parameters (CL/F, V_C_/F, MTT, relative bioavailability (F_rel_), and fa_1_). The covariates that displayed a correlation with a parameter were chosen for further analysis.

The significance of the relationships were investigated using the adjusted adaptive least absolute shrinkage and selection operator (AALASSO) [15, 16], as implemented in PsN [17] (version 4.7.0; Department of Pharmaceutical Biosciences, Uppsala University). LASSO is a penalized regression method, here used for covariate selection. In this method, a full covariate model is used, where all covariates are standardized to have zero mean and standard deviation one, and where the sum of all parameter-covariate coefficients is restricted to be smaller than a tuning parameter, *t*. The *t* value determines the size of the model and is estimated through cross-validation, where the dataset is split into, in this case, five groups. Model selection and estimation is made on four of the five groups (pooled) while the prediction error is calculated on the fifth group. This is repeated five times, to give, for a certain *t* value, a cross-validation estimate of the prediction error, where the appropriate *t* value is the one with the lowest prediction error. In contrast to the ordinary LASSO method, the AALASSO incorporates the ratio of the standard error of the maximum likelihood (ML) estimator to the ML coefficient as the initial weight and has shown to have a better predictive performance with a low number of subjects and highly correlated covariates. The data split, during cross-validation, was made on study association to preserve the relative proportions in the cross-validation datasets. The final covariates tested were CD dose on CL/F_LD_, age on CL/F_LD_, CL/F_CD_, F_rel,LD_, HY on CL/F_LD_, CL/F_CD_, F_rel,LD_, study association on CL/F_LD_, CL/F_CD_, F_rel,LD_, sex on MTT_2,CD_ (CD mean transit time between the second dose compartment and the central compartment), and years with LD treatment on CL/F_LD_. The covariates were included according to a linear covariate-parameter correlation:

$$ {P\theta}_i= TVP\theta \times \Big(\ 1+\left({P\theta}_{coeff}\times \left( COV-\overline{COV}\right)\right) $$

where *Pθ*_*i*_ is the individual parameter value, *TVPθ* is the typical parameter, *Pθ*_*coeff*_ is the corresponding coefficient representing the fractional change in *TVPθ*, *COV* is the covariate (0 or 1 for categorical covariates), and $\overline{COV}$ is the mean of the covariate.

Carbidopa is known to act as a peripheral dopa decarboxylase inhibitor, affecting the conversion of LD to dopamine. This effect was also evident in the initial covariate analysis, identifying the CD dose to have a significant effect on LD CL/F. To further investigate the influence of CD plasma concentrations on LD parameters, the LD and CD models were combined. In the combined model, the CD dose as well as the individual model-predicted CD concentration was investigated to influence CL/F_LD_, with a linear (as described above) or a non-linear covariate-parameter correlation:

$$ {P}_{\theta }=\frac{TVP\theta}{1+\left(\frac{COV}{P_{\theta, Inter}}\right)} $$

where *TVPθ* is the typical value of apparent levodopa clearance, *COV* is the covariate representing CD dose or CD concentration, and *Pθ*_*INTER*_ is the interaction factor representing the potency of CD as a competitive inhibitor. After investigating and including the effect of CD on LD PK, the AALASSO was repeated.

### Data analysis

The LD/CD measurements that were below limit of detection (5.6%) were handled using the M6 method [18], where LOD/2 is assigned to the first value and subsequent samples below LOD were deleted. The population PK model was developed using non-linear mixed effect modeling software NONMEM [19] (version 7.3; Icon Development Solutions, Ellicott City, MD, USA, 2009) using the first-order conditional estimation method (FOCE) with INTERACTION and a user-defined model (ADVAN6 NONMEM Subroutine). PsN [17] (version 4.7.0; Department of Pharmaceutical Biosciences, Uppsala University) was used for running models, simulating data, and testing covariate-parameter relations. R [20] (version 3.2.3; R Foundation for Statistical Computing) and Xpose [17] (version 4.5.0; Department of Pharmaceutical Biosciences, Uppsala University) were used for data management and graphical evaluation.

### Model evaluation

Models were evaluated by scientific plausibility, the objective function value (OFV), goodness-of-fit plots, parameter precision, and prediction-corrected visual predictive checks (pcVPCs, 1000 samples). The pcVPC was used to normalize for variability in independent variables, e.g., dose and bodyweight, in the graphical display of the predictive performance of the model [21]. The OFV, which approximates − 2 log(likelihood) of the data given the model, was utilized in likelihood ratio testing to compare nested models (significance level 0.05, corresponding to ∆OFV of 3.84 for 1 degree of freedom). Parameter uncertainty on model parameters was calculated with the sampling importance resampling (SIR) procedure [22]. The final levodopa model parameter estimates were externally validated with data from an open-label, multiple-dose microtablet study in ten healthy subjects (Study 3) [3]. The data from this study was not used in the model development, only for external model evaluation.

## Results

### Base model

The final base model for CD and LD was a one and two-compartment model respectively, with parallel absorption compartments including transit compartments describing absorption delay (∆OFV of 995 and 240 for CD and LD respectively compared to a model with a single absorption compartment). The final parameter estimates are presented in Table 2. The models were parameterized in terms of the fraction absorbed from the fast absorption compartment (fa_1_), absorption compartment-specific mean transit-time (MTT_1_ and MTT_2_), volume of the central (V_C_/F) and peripheral (V_P_/F, only of LD) compartment, clearance (CL/F), and inter-compartmental clearance (Q/F, only for LD). The optimal number of transit compartments were five and six for levodopa, and three and ten for carbidopa. The residual error model was a combined proportional and additive error model separately estimated for the different drugs (CD and LD) and populations (healthy volunteers and PD patients). For LD, the additive error was estimated to a low value for PD patients and therefore fixed to 0. When the LD and CD models were combined, it was estimated that 28.5% of the total residual error (proportional and additive combined) was shared between LD and CD. Inter-individual variability was estimated for relative bioavailability (F_rel_), fa_1_, MTT_1_, MTT_2_, CL/F, and V_C_/F (only for LD). The pre-dose concentration was estimated to 0.12 and 0.026 μg/mL for LD and CD, respectively, both associated with high inter-individual variability (180 and 90% for LD and CD respectively).

**Table 2:** Parameter estimates for the final LD/CD population pharmacokinetic model and results from the SIR evaluation

Parameter	Levodopa (%RSE) ^§^ [%Shrinkage]	Carbidopa (%RSE) ^§^ [%Shrinkage]	Levodopa SIR (%RSE) ^§^ [95% CI^¶^]	Carbidopa SIR (%RSE) ^§^ [95% CI^¶^]	Levodopa^×^ (%RSE) ^§^ [%Shrinkage]	Carbidopa^×^ (%RSE) ^§^ [%Shrinkage]
CL/F (L/min)*(WT/70)^0.75^	1.31^†^ (10.7)	1.05 (8.46)	1.31^†^ (10.6) [1.08–1.63]	1.05 (8.57) [0.881–1.23]	1.21^†^ (10.1)	1.05 (8.34)
V_C_/F (L)*(WT/70)	45.4 (9.85)	168 (8.50)	46.0 (9.17) [38.6–55.2]	167 (8.13) [142–195]	45.5 (9.91)	168 (8.58)
Q/F (L/min)*(WT/70)^0.75^	0.667 (15.7)	–	0.667 (11.2) [0.548–0.837]	–	0.661 (15.3)	–
V_P_/F (L)*(WT/70)	44.9 (5.90)	–	44.8 (6.01) [40.0–50.3]	–	45.1 (5.88)	–
MTT_1_ (min)	16.1 (6.15)	34.6 (6.20)	16.1 (6.05) [14.3–18.2]	34.7 (5.73) [30.9–38.7]	16.1 (6.11)	34.6 (6.05)
MTT_2_ (min)	86.2 (6.39)	121 (6.01)	85.9 (4.69) [78.1–94.4]	121 (5.27) [109–134]	86.5 (6.27)	120 (5.40)
F_REL_	1 FIX	1 FIX	1 FIX	1 FIX	1 FIX	1 FIX
fa_1_^‡^	0.749 (5.35)	0.570 (7.10)	0.749 (4.67) [0.678–0.816]	0.570 (6.61) [0.50–0.65]	0.747 (5.36)	0.570 (6.73)
Pre-dose concentration (μg/mL)	0.120 (42.3)	0.0256 (38.8)	0.129 (45.9) [0.0542–0.268]	0.0256 (32.5) [0.01–0.04]	0.120 (42.3)	0.0255 (39.5)
INTER_CL/F-CDAMT_ (mg)	86.7 (36.3)	–	94.6 (32.8) [52.0–161.5]	–	119.1 (37.3)	–
CL/F-AGE	–	− 0.0119 FIX	–	− 0.0119 FIX	–	− 0.0114 (19.8)
CL/F-HY	− 0.0616 FIX	0.0141 FIX	− 0.0616 FIX	0.0141 FIX	− 0.0967 (27.0)	–
F_REL_-AGE	0.00172 FIX	–	0.00172 FIX	–	–	–
MTT_2_-SEX	–	0.178 FIX	–	0.178 FIX	–	–
IIV_CL/F_	15.0 (31.8) [25.8]	20.9 (16.0) [18.4]	16.4 (27.6) [8.45–23.7]	21.8 (19.8) [14.5–29.6]	14.6 (32.0) [26.8]	21.0 (16.1) [18.4]
IIV_VC/F_	39.5 (22.8) [17.3]	–	41.2 (17.4) [28.2–53.9]	–	38.9 (22.1) [17.0]	–
IIV_MTT1_	34.5 (13.8) [6.49]	31.9 (10.6) [6.55]	35.3 (14.3) [26.6–45.9]	32.9 (12.7) [25.6–40.9]	34.4 (13.7) [6.34]	32.1 (10.3) [6.39]
IIV_MTT2_	15.2 (34.7) [18.0]	27.2 (19.5) [3.98]	16.5 (28.5) [8.67–24.5]	27.6 (15.2) [20.6–36.2]	15.3 (33.5) [17.7]	29.2 (12.4) [3.47]
IIV_fa1_^‡^	99.6 (15.8) [10.0]	81.2 (19.2) [9.53]	104 (17.0) [77.6–139]	84.7 (16.4) [62.9–112]	99.1 (15.6) [10.5]	80.5 (17.0) [9.54]
IIV_Frel_	28.3 (20.6) [4.89]	48.1 (9.69) [1.60]	28.7 (14.3) [21.2–36.4]	49.5 (12.7) [38.5–61.8]	28.8 (19.7) [4.55]	48.4 (9.63) [1.56]
IIV_Pre-dose concentration_	180 (11.3) [28.3]	89.6 (24.9) [59.3]	187 (15.1) [138–239]	102 (36.3) [55.6–156]	1.80 (11.3) [28.3]	90.1 (24.9) [59.2]
Proportional error (%)						
Healthy subjects	15.0 (11.3)	7.70 (14.1)	15.0 (6.14) [13.5–17.1]	7.70 (11.4) [6.08–9.49]	15.0 (11.3)	7.66 (13.8)
Patients	7.02 (14.6)	3.86 (35.3)	7.07 (6.51) [6.22–8.04]	3.86 (20.2) [2.21–5.27]	7.03 (14.5)	3.89 (33.8)
Additive error (μg/mL)						
Healthy subjects	0.00406 (16.6)	0.00415 (14.2)	0.00406 (9.15) [0.00343–0.00491]	0.00418 (5.85) [0.00372–0.00468]	0.00407 (16.5)	0.00415 (14.3)
Patients	–	0.00684 (28.3)	–	0.00693 (13.5) [0.00526–0.00880]	–	0.00677 (27.5)

^†^Per milligram carbidopa for typical individual with 0 mg carbidopa and mean HY 1.53; ^‡^Logit transformed; ^§^NONMEM point estimate and the associated % relative standard error (% RSE, reported on the approximate standard deviation scale (SE/variance estimate)/2). ^¶^The median and 95% confidence interval (2.5th and 97.5th percentiles). ^**×**^With unfixed parameter-covariate coefficients (relationships with little influence were removed). *CI*, confidence interval; *CDAMT*, carbidopa dose; *HY*, Hoehn and Yahr; *IIV*, inter-individual variability (CV%); *SIR*, sampling importance resampling

### Covariate model

Carbidopa dose was found to have a significant effect in the initial covariate analysis, with a decrease in OFV of 24 when a linear relationship was used and a decrease in OFV of 30 when described with a non-linear relationship between carbidopa dose and CL/F_LD_. The drop in OFV was less than 3.84, i.e., not significant, when individual model-predicted CD concentration was added as a covariate on LD CL/F. The parameter describing the interaction (INTER_CL/F,LD-CDAMT_) was estimated to 86.7 mg (Table 2), which results in a LD CL/F estimated to 48.5 L/h with 50 mg CD and 40 L/h with 75 mg CD, for a 70-kg subject. The relationship should not be extrapolated outside the dose range studied.

After including the effect of CD dose on LD CL/F, a final covariate search was performed using the AALASSO method. To reduce run-time, this step was performed on separate models for CD and LD. For CD, the covariate-parameter relations selected by AALASSO were age and HY on CL/F (coefficients − 0.012 and 0.014 respectively) and sex on the mean transit time for the second peak (MTT_2,CD_, coefficient 0.178). The CL/F_CD_ was estimated to decrease by 28% between the age of 60 and 80 years. A disease progression from HY 1 to 4 was estimated to result in a very modest and clinically insignificant increase of 0.4%. The MTT_2,CD_ was estimated to be 19% (22 min) longer for women. The covariate-parameter relations selected by AALASSO for LD were HY on CL/F (coefficient − 0.062) and age on F_rel,LD_ (coefficient 0.002). This suggests an 18% decrease in CL/F_LD_ related with a disease progression from HY 1 to 4. Although the increase in F_rel,LD_ with age was found to be significant, the effect was very modest, with a decrease of 3.4% between the age 60 to 80 years. Finally, models with and without the inclusion of the clinically significant parameter-covariate relationships obtained from the AALASSO were re-estimated. Including age on CL/F_CD_ resulted in a drop in OFV of 24, and HY on CL/F_LD_ resulted in a drop in OFV of 7.4.

The observed and model-predicted individual plasma concentrations for the 36 subjects included during model development are shown in the supporting material (Online Resource 2). The concentration-time profiles, including the double-peak phenomena, are well captured in the subjects for both CD and LD.

**Fig. 1:**
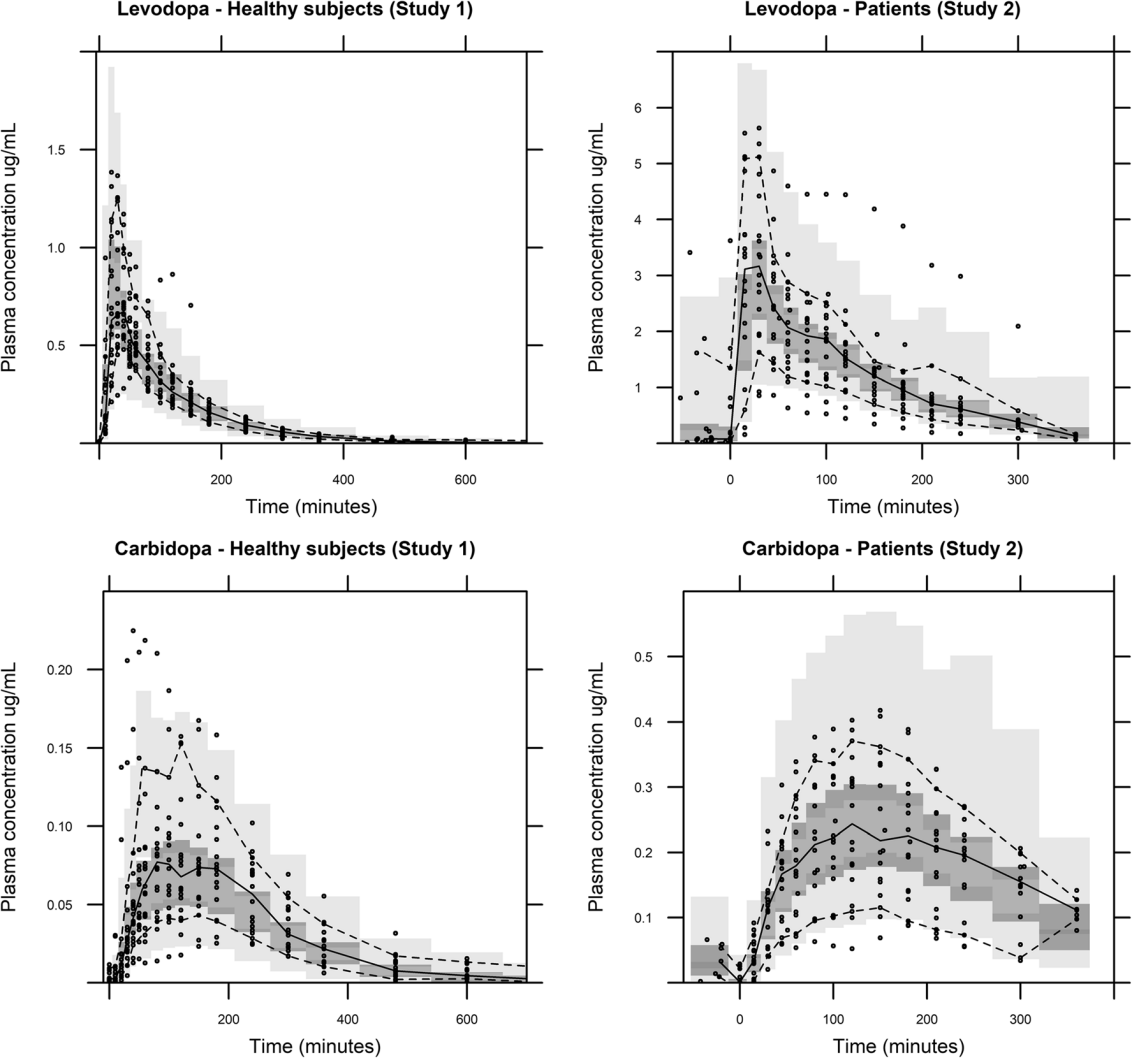
Prediction corrected visual predictive check (1000 samples) of the continuous data for the LD/CD dispersible microtablets after covariate model selection stratified on healthy volunteers and PD patients and LD and CD. The solid line is the median of the observed data. The dashed lines represent the observed 10th and 90th percentile of the observations. The top and bottom light gray areas are the 5th and 95th confidence intervals for 10th and 90th percentiles of the simulated data. The middle dark gray area is the 5th and 95th confidence interval for the median of the simulated data

**Fig. 2:**
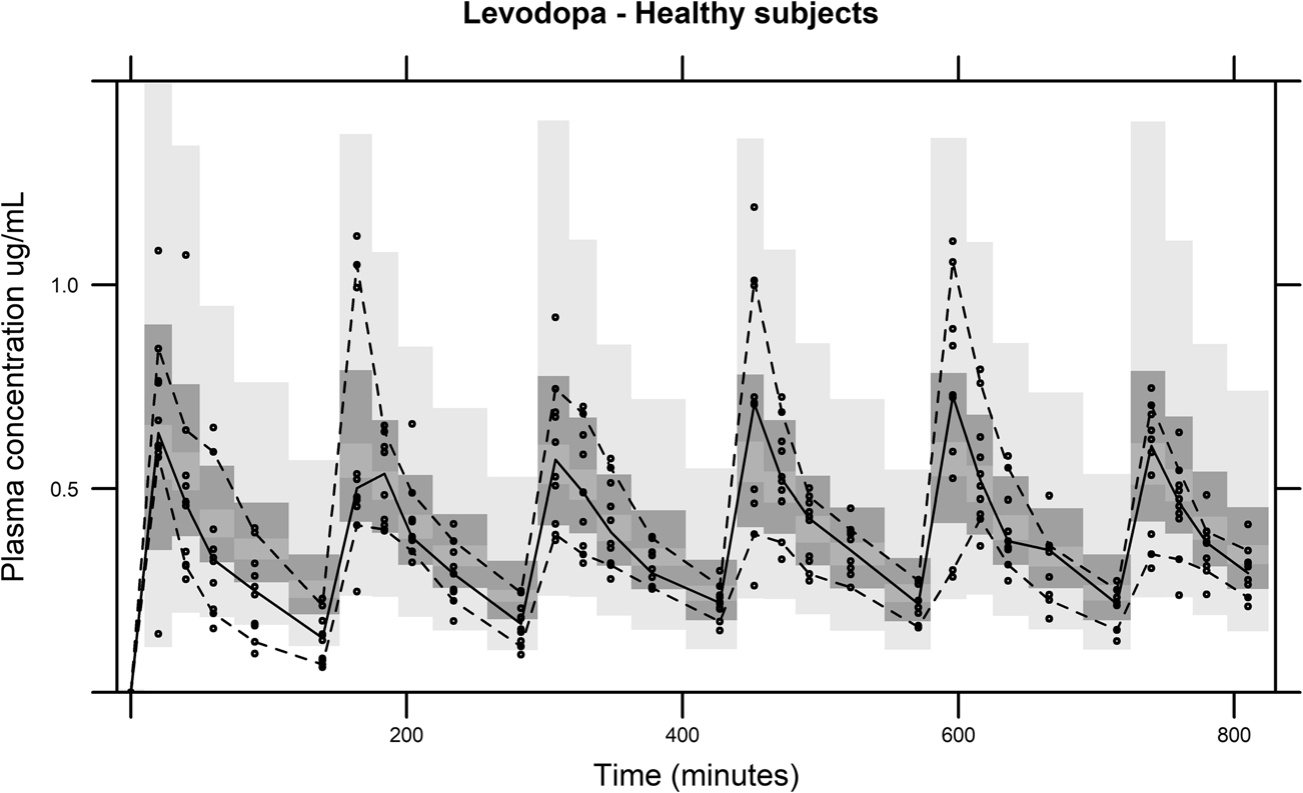
External evaluation of the predicitive performance of the final levodopa model with covariates (1000 samples) based on data from Study 3. The solid line is the median of the observed data. The dashed lines represent the observed 10th and 90th percentile of the observations. The top and bottom light gray areas are the 5th and 95th confidence intervals for 10th and 90th percentiles of the simulated data. The middle dark gray area is the 5th and 95th confidence interval for the median of the simulated data

The pcVPC, for the final models stratified by study association, is shown in Fig. 1. The median plasma concentrations for both populations were well captured; however, the early LD peak plasma concentration is slightly overestimated for the patient population. The final LD model was used for prediction of data from an external dataset that was not used in the model development process (Fig. 2). In accordance with the procedure for Study 1 and 2, the total CD dose administered during the study period was used to describe the CD/LD interaction. The model predictions capture the observations relatively well, especially at later time points. However, the model is over-predicting the observed LD concentrations in the beginning of the study, indicating initial time-dependent changes in LD PK, not optimally captured by the model

## Discussion

The present analysis characterized the pharmacokinetics of LD and CD when administered as dissolved microtablets to PD patients and healthy subjects, and investigated the influence of CD dose and plasma concentration on LD pharmacokinetic parameters as well as other covariate-parameter relationships to describe inter-individual PK differences. Levodopa PK has previously been described with both one- and two-compartmental models [23–25]. A two-compartment model was found to provide the best description of LD in this case (OFV reduced by 147.4 compared to a one-compartment model). The estimated LD CL/F, with 95 mg of CD, for a patient with HY stage 3, was here estimated to 34 L/h/70 kg, which is close to the CL/F_LD_ of 37 L/h/70 kg reported for advanced PD patients where the majority (24/30) were at HY stage 3 who also received individual doses of LD/CD (mean CD dose of 96 mg) [26]. The total LD volume of distribution (V_Total_/F) was estimated to 90 L. Previously reported V_Total_/F varies between 39 to 131 L, probably reflecting both variations in study population and analysis technique [23, 24, 26, 27]. Carbidopa was best described with a one-compartment model, where CL/F was estimated to 63 L/h and V_C_/F to 168 L, corresponding to a half-life of 111 min for the typical individual of 70 kg and 49 years of age.

The LD and CD model incorporates a description of the double-peak profile that may occur with LD/CD administrations. The mechanism behind the presence of double peaks is not entirely understood, but is theorized to occur due to metabolized LD in the gastrointestinal tract, causing a cessation in gastric emptying [28, 29]. The LD double-peak phenomenon has to our knowledge previously only been modeled by Ogungbenro et al. (2015). Their suggested semi-mechanistic model was however supported by available scintigraphy data and paracetamol PK data, and was too complex for the data on hand, which when implemented resulted in unidentifiable parameters. The inclusion of parallel absorption compartments was found as the most appropriate model for the description of this phenomenon (Online Resource 2).

A non-linear decrease of CL/F_LD_ was found with increased carbidopa dose. The metabolism of LD mainly occurs during first-pass metabolism [30], and an animal study suggested that it occurs mainly in the intestine [31], which could be a reason to why CD plasma concentration was not found to explain the variability in CL/F_LD_. Jorga et al. (2000) [23] found increasing levodopa dose to significantly decrease CL/F_LD_. Levodopa was in that study co-administered with a dopa decarboxylase-inhibitor (carbidopa or benserazide) in a 1:4 ratio. Further, a non-linear increase in AUC of levodopa has been previously described, when administered alone; however, the doses administered were higher (between 3.8 and 15.4 mg LD/kg) than in our study [31]. In our analysis, it is not possible to dissociate if it is the dose of CD, LD, or a combination of both that causes a decrease in CL/F_LD_, because all patients received both drugs in a 1:4 ratio. It may be of interest to investigate whether carbidopa could be given differently compared to today’s 1:10 and 1:4 formulations with oral levodopa treatment [32]. The external validation of the model was performed with data from a multiple-dose study in healthy subjects, where patients received lower doses LD/CD every 2.4 h [3]. The plasma concentration (Fig. 2) after first dose is slightly over-predicted by the model. A reason for this could be that CL/F_LD_ is adjusted with the total administered CD dose, instead of a cumulative amount over time.

For investigation of influential covariates, the AALASSO method was chosen since it has shown to perform better with small dataset and with highly correlated covariates [16], which was the circumstance in this case. The advantage of the method is that it tests all relationships simultaneously and does not rely on a user-specified *p* value; however it may, as in this case, add some covariates that only have a modest effect on the parameter values. In the analysis, age, sex, and HY were found to separately improve the prediction of the data. The disease severity (HY stage) was in this analysis found to be the most influential covariate affecting the apparent CL of LD. Previous studies have found LD CL/F to be related to sex, creatinine clearance, LD dose, and age [23, 33]. Hoehn and Yahr score may in our analysis be a representation of a combination of covariates (e.g., age and years with LD treatment) all contributing to the total effect observed. The relative bioavailability (F_rel,LD_) was here found to be slightly increased with age (3.4% between the age 60 and 80 years). The effect is probably not clinically relevant. For CD, age was identified as a significant covariate on CL/F_CD_. The estimated half-life for the mean age of the patient population (71.4 years) was 155 min, which is somewhat lower than the mean half-life estimated with non-compartmental analysis (171 ± 37 min) [6]. Sex was found as a significant covariate on MTT_2,CD_, with the second plasma concentration-time peak appearing 22 min later for women. This indicates a slower gastric emptying rate for women, compared to men. Such a sex-related difference has been observed previously in studies where gastric emptying has been specifically investigated [34, 35]. On the parameters found to be influenced by covariates in the graphical analysis (F_rel,LD_, CL/F_LD_ and CL/F_CD,_ MTT_2,CD_), study association was included as a covariate on all but MTT_2,CD._ Interestingly, it was not added on any of the parameters in the AALASSO, indicating other covariates to have a higher predictive value.

It is important to note that both studies included in model development had few subjects and the data consisted only of single-dose administration data. The results need to be confirmed in a larger population study, mainly with patients. For future development of the model, inclusion of multiple-dose studies in patients should be added and the model should be coupled with pharmacodynamic data, assessed both by physicians and by patients with the dose-dispenser diary, to ensure optimal dose suggestions for individual patients.

In summary, a population PK model was developed that adequately describe the PK of LD and CD, following LD/CD microtablet administration in healthy subjects and Parkinson’s disease patients. In our study, the covariates identified that could have a clinical significance were CD dose and HY on LD CL/F, and age on CD CL/F. The developed model may in the future be used for individualized dose selection, utilizing the flexibility offered by the microtablets.

## Electronic supplementary material

ESM 1 (PDF 125 kb)

ESM 2 (PDF 590 kb)
