# Supplementary material for: Population pharmacokinetics of levodopa/carbidopa microtablets in healthy subjects and Parkinson’s disease patients
Source: Eur J Clin Pharmacol. 2018 Jun 7;74(10):1299–307. doi: 10.1007/s00228-018-2497-2 (PMC6132549; doi:10.1007/s00228-018-2497-2)
Supplement: Supplementary file 1 — (PDF 125 kb) [file 228_2018_2497_MOESM1_ESM.pdf]

# Population pharmacokinetics of levodopa/carbidopa microtablets in healthy subjects and Parkinson's disease patients

European Journal of Clinical Pharmacology

Marina Senek[1,2] PhD, Dag Nyholm[1] MD PhD, Elisabet I Nielsen[2] PhD

[1]Department of Neuroscience, Neurology, Uppsala University, Sweden[1]

[2]Department of Pharmaceutical Biosciences, Uppsala University, Sweden[2]

Marina Senek, corresponding author, ORCID 0000-0003-0302-6946

Department of Neuroscience, Neurology

Uppsala University

Akademiska Sjukhuset/Uppsala University Hospital

751 85 Uppsala, Sweden

E-mail: marina.senek@neuro.uu.se

[1] Uppsala University Hospital, 751 85 Uppsala, Sweden
[2] Uppsala biomedicinska centrum BMC, Husarg. 3, Box 591, 751 24 UPPSALA

## Bioanalysis

The methods used were validated in agreement with the ICH Validation of Analytical Procedures[1] and the Guideline on Bioanalytical Method Validation[2].

### Levodopa/carbidopa microtablets in healthy subjects (Study 1 and 3)[3,4]

Blood samples were immediately centrifuged (10 min, 3100 rpm) after collection in EDTA tubes containing 143 IU of heparin. The samples were stored frozen (-75°C) until analysis after addition of 50 µL 10% sodium metabisulfite solution. The plasma samples were thawed, and after protein precipitation, the plasma concentrations of levodopa and carbidopa were determined (limits of quantification (LOQ) were 12 and 15 ng/mL respectively) (Supplementary Table 1).

### Levodopa/carbidopa microtablets in patients (Study 2)[5]

Collected blood samples (in EDTA tubes) were stored on ice, centrifuged within 1 hour (20 min, Sorvall SL50T, 3900 rpm), and stored frozen at −80 °C until analysis. The plasma samples were thawed, and after protein precipitation, the plasma concentrations of levodopa and carbidopa were determined (limits of quantification (LOQ) were 10 and 20 ng/mL respectively) (Supplementary table 1). The analysis was conducted at The Department of Pharmacology, University of Gothenburg, Sweden.

Supplementary Table 1. Bioanalysis equipment

| | **Study 1 and 3** | **Study 2** |
|---|---|---|
| **Chromatography** | HPLC (2250 Bischoff) | HPLC (Dionex Ultimate 3000 pump) |
| **Detector** | Coulochem II multi-electrode detector ESA (Chelmsford, Mass) | Waters 450 amperometric detector |
| **Column** | C18-AQ particle size 5 µm guard column (Reprosil-Pur) | C18 reverse phase column (Onyx) 2.0 mm x 200 mm |
| **Mobile phase** | 100-mmol/L sodium dihydrogen orthophosphate, pH 3.0, containing 0.5-mmol/LOSA, 1-mmol/L EDTA, and 7% methanol | 50 mmol/L phosphate buffer, pH 2.88 with EDTA 10 mg/L, methanol 4.0%, acetonitrile 1.5% and 1-octanesulphonic acid 100 mg/L |
| **Tray cooling** | +5 °C | +4 °C |

HPLC, High performance liquid chromatography; UPLC, Ultra performance liquid chromatography.

## References


1. ICH Topic Q 2 B, Validation of Analytical Procedures: Methodology, Note for guidance on validation of analytical procedures (CPMP/ICH/281/95).

2. EUROPEAN MEDICINES AGENCY. Guideline on bioanalytical method validation. *Committee for Medicinal Products for Human Use (CHMP).* 2011.

3. Nyholm D, Lewander T, Gomes-Trolin C, et al. Pharmacokinetics of levodopa/carbidopa microtablets versus levodopa/benserazide and levodopa/carbidopa in healthy volunteers. Clin Neuropharmacol. 2012;35:111–117.

4. Nyholm D, Ehrnebo M, Lewander T, et al. Frequent administration of levodopa/carbidopa microtablets vs levodopa/carbidopa/entacapone in healthy volunteers. Acta Neurol Scand. 2013;127:124–132.

5. Senek M, Aquilonius S-M, Askmark H, et al. Levodopa/carbidopa microtablets in Parkinson's disease: a study of pharmacokinetics and blinded motor assessment. Eur J Clin Pharmacol. 2017;73:563–571.
