# Supplementary material for: Population pharmacokinetics of levodopa/carbidopa microtablets in healthy subjects and Parkinson’s disease patients
Source: Eur J Clin Pharmacol. 2018 Jun 7;74(10):1299–307. doi: 10.1007/s00228-018-2497-2 (PMC6132549; doi:10.1007/s00228-018-2497-2)
Supplement: Supplementary file 2 — (PDF 590 kb) [file 228_2018_2497_MOESM2_ESM.pdf]

Population pharmacokinetics of levodopa/carbidopa microtablets in healthy subjects and Parkinson's disease patients

European Journal of Clinical Pharmacology

Marina Senek[1,2] PhD, Dag Nyholm[1] MD PhD, Elisabet I Nielsen[2] PhD

[1]Department of Neuroscience, Neurology, Uppsala University, Sweden[1]

[2]Department of Pharmaceutical Biosciences, Uppsala University, Sweden[2]

Marina Senek, corresponding author, ORCID 0000-0003-0302-6946

Department of Neuroscience, Neurology

Uppsala University
Akademiska Sjukhuset/Uppsala University Hospital

751 85 Uppsala, Sweden

E-mail: marina.senek@neuro.uu.se

---

[1] Uppsala University Hospital, 751 85 Uppsala, Sweden

[2] Uppsala biomedicinska centrum BMC, Husarg. 3, Box 591, 751 24 UPPSALA

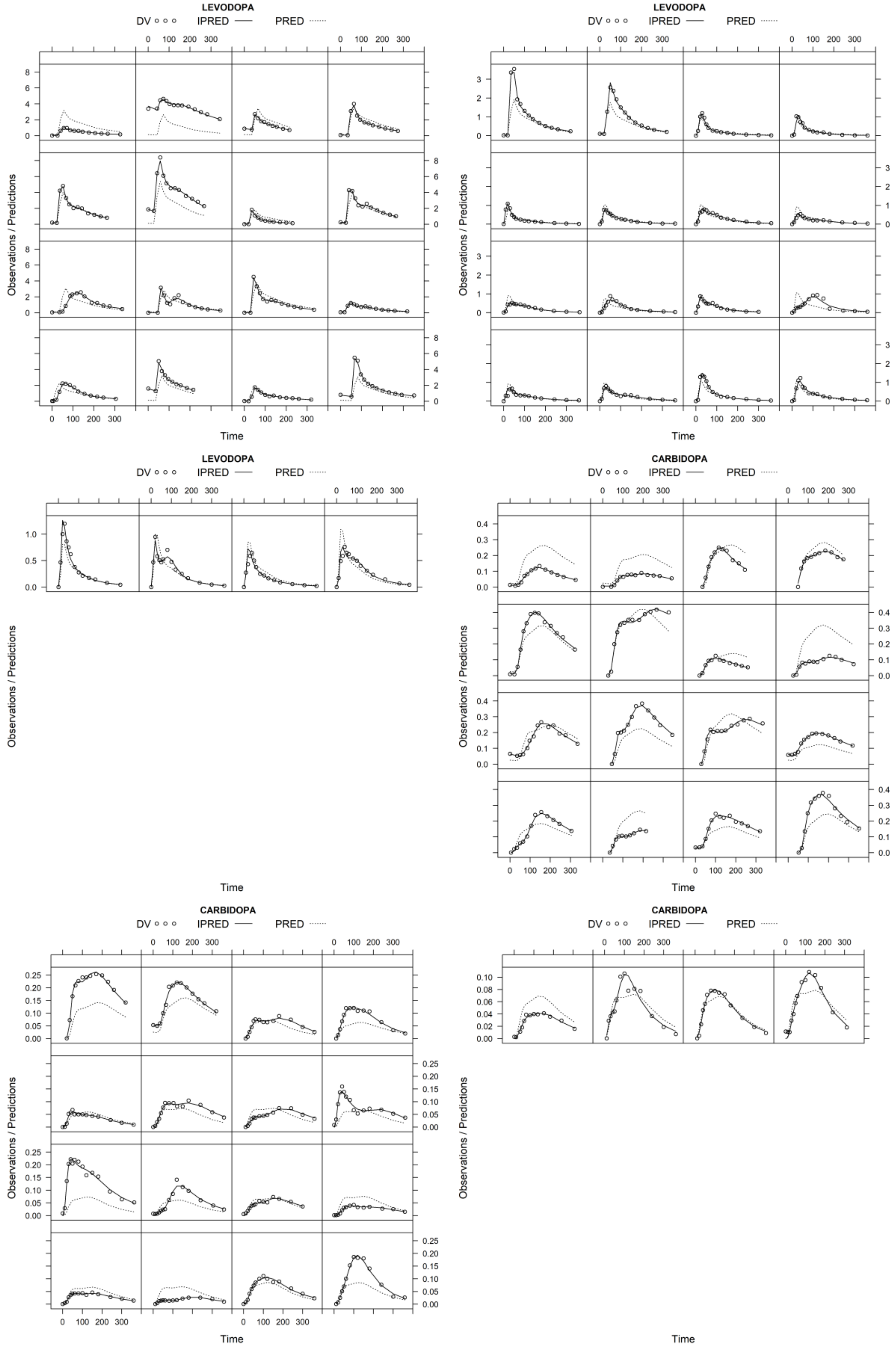

Online Resource 2. Individual plots of levodopa and carbidopa plasma concentration over time. The points are the observed values, solid lines are the individual model predictions and the dashed lines represent the population prediction.
